# Sensitive Assay for Carvedilol in Tablets and Spiked Human Plasma Using a Flow-Injection Chemiluminometric Method

**Published:** 2007-06

**Authors:** Nawal Al Arfaj, Heba H. Abdine, Maha A. Sultan

**Affiliations:** 1 *Department of Chemistry, College of Science, Women Student-Medical Studies and Sciences Sections, King Saud University, P.O. Box 22452, Riyadh 11495, Saudi Arabia;*; 2 *Department of Pharmaceutical Chemistry, College of Pharmacy, Women Student-Medical Studies and Sciences Sections, King Saud University, P.O. Box 22452, Riyadh 11495, Saudi Arabia*

**Keywords:** carvedilol, chemiluminescence, flow injection, Tris (2, 2’-bipyridyl) ruthenium (II), KMnO_4_, tablets, human plasma

## Abstract

A simple and sensitive chemiluminometric method using flow injection (FI) is developed for the determination of carvedilol, based on the reaction of carvedilol with tris (2, 2’-bipyridyl) ruthenium (II), and KMnO_4_ in sulfuric acid medium. Under the optimum conditions; the chemiluminescence (CL) intensity is a linear function of carvedilol concentration over the range of 0.04–1.0 µg ml^-1^ (9.8 × 10^-8^ - 2.5 × 10^-6^ mol L^-1^) with a detection limit (S/N=3) of 0.025 µgml^-1^ (6.2 × 10^-8^ mol L^-1^). The relative standard deviation of the proposed method calculated from 10 replicate injections of 0.4 µg ml^-1^ carvedilol is 0.95%. The sample throughput is 90 samples h^-1^. The method is applied successfully to the determination of carvedilol in tablets dosage form and spiked human plasma.

## INTRODUCTION

**Figure 1:**
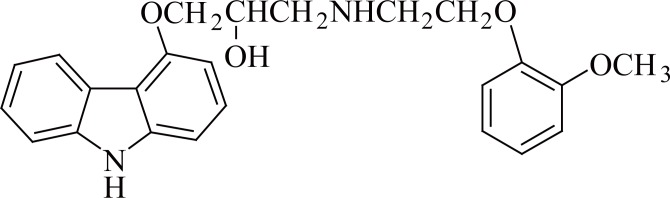
Structure of carvedilol.

Carvedilol (±)-1-(carbazol-4-yloxy)-3-[(2-(O-methoxyphenoxy)ethyl)amino)-2-propanol is a non-cardioselective β-blocker (1) (Fig. 1). It also has vasodilating properties. It is used in the management of hypertension and angina pectoris, and as an adjunct to standard therapy in symptomatic heart failure (1, 2).

The European Pharmacopoeia (3) describes a non-aqueous titration procedure for the determination of carvedilol in bulk.

Several methods are described for the determination of carvedilol in biological fluids including high performance liquid chromatography (4-9) and capillary electrophoresis (10-12). Few methods are reported for the analysis of carvedilol in tablets including fluorimetry (13, 14) and differential pulse voltammetry (15).

Although most of these methods are specific, their applications are limited due to the high commercial price. Chemiluminescence (CL) has received much attention as an attractive method for analytical application because of its high sensitivity, high selectivity, and small amount of chemical consumption, cost effectiveness, simple sample preparation and instrumentation (16). Only one procedure is reported in the literature for the determination of carvedilol in pharmaceutical dosage forms by using flow injection with chemiluminescence detection (17). The procedure is based on the inhibition of the chemiluminescence response resulting from oxidation of luminol by hypochlorite in a multi-pumping flow system.

[Ru(bipy)_3_^2+^] is used as the basis of chemiluminescence detection of a wide range of compounds after oxidation to [Ru(bipy)3^3+^], which is then followed by reduction with an analyte species with the subsequent emission of light (18, 19).

The present study describes a flow injection chemiluminometric procedure for the determination of carvedilol. The method is based on the chemiluminescence reaction of the drug with [Ru(bipy)_3_^2+^] and potassium permanganate in sulfuric acid medium. Although, carvedilol has been determined using a multi-pumping flow system with the detection of the CL generated from the luminol system in presence of hypochlorite (17), the proposed method is easier to implement for routine analysis in pharmaceutical control using a simpler equipment. This straight forward method has shown its usefulness in the analysis of carvedilol in tablets and spiked human plasma.

## EXPERIMENTAL

### Apparatus and manifold

**Figure 2:**
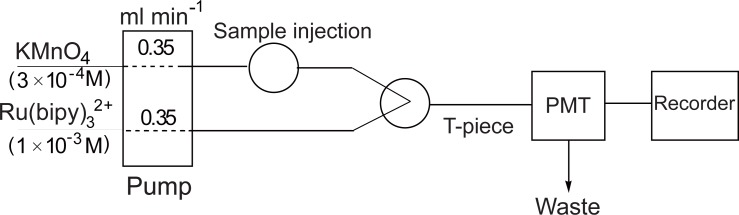
Flow-injection manifold for CL determination of carvedilol.

The flow system used for the determination and CL detection of carvedilol is shown schematically in Fig. 2. A Gilson minipuls 3M P4 peristaltic pump (two channels, variable speed) was used to drive the carrier and the reagent streams through the flow system. Each stream was pumped at a constant flow rate using PTFE tubing (0.8 mm i.d.).

The drug solution (100 µl) was injected through the sample injection valve, which allows the mixing of the sample with an acidified 3 × 10^-4^ M KMnO_4_ solution and then combined with 1 × 10^-3^ M [Ru(bipy)_3_^2+^] solution just before the detector. The emitted light intensity was measured by a photomultiplier tube (PMT, THORN EMI 9789QB) which was operated at 1100 V. The PMT voltage was provided by a stable power supply (THORN EMI, Model PM 288BN).

The signal was recorded by a Yokogawa Model 3021 Recorder (Yokogawa, Japan). Peak height was measured for each signal and expressed as a voltage output of the photomultiplier tube.

### Reagents

All the reagents used were of analytical reagent grade and the solutions were prepared with doubly distilled water. The following reagents were used: aqueous potassium permanganate (Fluka, UK), solution of 3 × 10^-4^ M was prepared in 0.8 M sulfuric acid (Reidel-deHaen, Germany); aqueous [Ru(bipy)_3_^2+^] (Aldrich, Chem. Co.) solution of 1 × 10^-3^ M was prepared by dissolving [Ru(bipy)_3_^2+^] hexahydrate in distilled water.

### Materials

Pure drug samples, (99.8%), were kindly supplied by Saudi Pharmaceutical Industries & Medical Appliances Corporation, Al-Qassim Pharmaceutical Plant, and Saudi Arabia (Dilatrend tablets (B.N.M 1003), each labeled to contain 25 mg carvedilol and manufactured by Saudi Pharmaceutical Industries under License of F. Hoffmann – La Roche Ltd, Basel, Switzerland by Roche S.P.A. Milan, Italy, were obtained from commercial sources.

Human plasma was obtained from King Khalid University Hospital, Blood Bank, King Saud University, and Riyadh and kept frozen until use after gentle thawing.

### Preparation of standard solution

Stock solution (1.0 mg ml^-1^) of carvedilol was prepared by dissolving 50.0 mg of carvedilol in methanol in a 50-ml measuring flask. The solution was protected from light and stored in a refrigerator at approximately 4°C, it remained stable for at least 2 weeks. Working standard solutions with carvedilol concentrations ranging from 0.04 to 1.0 µg ml^-1^ were daily prepared by methanol dilutions of the above stock solution.

### Recommended procedure for calibration

The FI manifold described in Fig. 2 was used. A 100 µl of drug solution was injected into a stream of acidified 3 × 10^-4^ M KMnO_4_ solution which was then combined with a stream of 1 × 10^-3^ M Ru(bipy)_3_^2+^ solution. The resulting peak height in mV was measured and plotted against drug concentration to obtain a calibration curve. Alternatively, the regression equation was derived.

### Procedure for pharmaceutical formulations

Ten tablets were weighed and finally powdered. A weighed amount of the fine powder equivalent to 10 mg of carvedilol was dissolved in methanol by sonication for 10 min. The solution was filtered into a 100 ml volumetric flask and the filtrate was diluted to volume with methanol. This solution, labeled to contain 100 µg ml^-1^ of drug, was analyzed by the FI-CL procedure described above for calibration, and a standard addition method was used. Nominal content of tablets was calculated either from a previously plotted calibration graph or by using the regression equation.

### Procedure for spiked human plasma

Different concentrations of standard carvedilol (0.1, 0.4 and 0.8 µg ml^-1^) were added to 1 ml drug free plasma in centrifuge tube. 5 ml of methanol was added and vortexed for 2 min. The tube was centrifuged for 5 min at 2600 rpm. The supernatant was transferred to another tube and evaporated under nitrogen at 50°C. 1 ml of methanol was added to the dry residue and the sample was dissolved by shaking for 30 s at 1500 rpm. Finally, the solution was centrifuged for 5 min at 2600 rpm and the supernatant was transferred to the polypropylene autosampler vial and was analyzed by the FI-CL procedure as described above for calibration. Nominal content of each plasma sample was calculated by using the regression equation for the drug in plasma previously calculated.

## RESULTS AND DISCUSSION

### Optimization of the flow injection system

**Manifold design**. A two-line manifold was used for the determination of carvedilol (Fig. 2). Maximum CL intensity was obtained when the sample was injected into a stream of acidified KMnO_4_ and then mixed with Ru(bipy)_3_^2+^ just before the detector.

**Effect of different oxidants.** Several oxidants such as K_2_Cr_2_O_7_, Ce (IV), H_2_O_2_, Fe (III) and KMnO_4_ were evaluated. It was found that KMnO_4_ produced the highest CL intensity.

**Figure 3:**
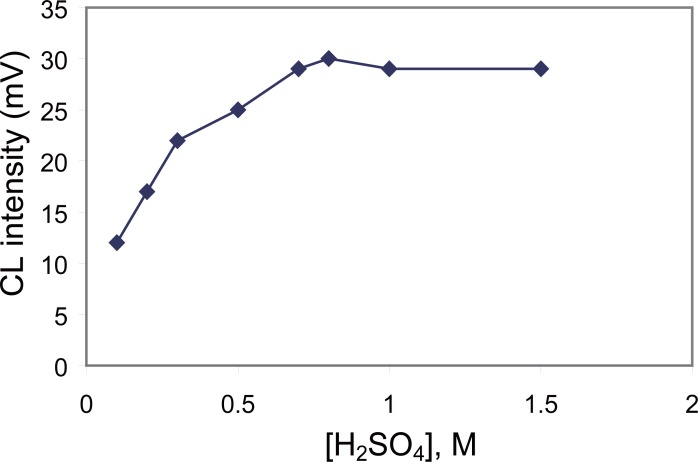
Effect of H_2_SO_4_ concentration as a diluent for KMnO_4_ on the CL intensity of carvedilol (0.4 µg ml^-1^).

**Effect of different concentrations of acids.** HCl, HNO_3_, H_3_PO_4_ and H_2_SO_4_ were used in different concentrations as diluent for KMnO_4_ solution to study their effect on the CL intensity. The results showed that the maximum CL signal was attained with H_2_SO_4_. The influence of different concentrations of H_2_SO_4_ from 0.1–1.5 M was studied. It was found that the CL intensity increased with increasing H_2_SO_4_ concentration and reached maximum value at 0.8 M, (Fig. 3). Thus, a concentration of 0.80 M was selected for this work.

**Figure 4:**
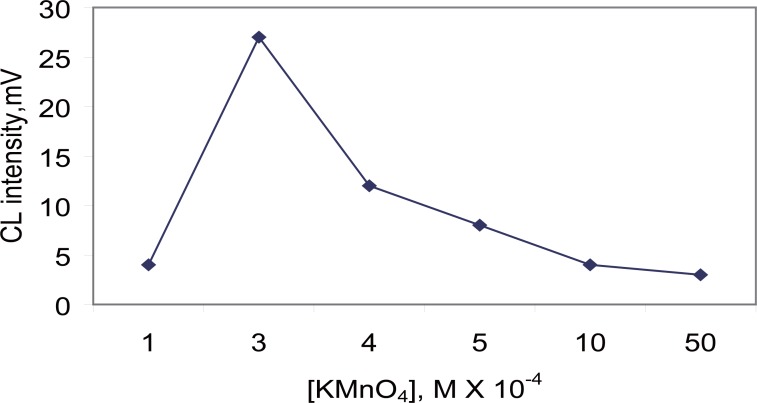
Effect of KMnO_4_ concentration on the CL intensity of carvedilol (0.4 µg ml^-1^).

**Effect of KMnO_4_ concentration.** The results of experiments showed that KMnO_4_ concentration dramatically influenced the CL signals. So the concentration of KMnO_4_ was studied ranging from 1 × 10^-4^ – 5 × 10^-3^ M. The peak height increased with raising KMnO_4_ concentration up to 3 × 10^-4^ M above which the CL intensity decreased sharply. Probably, because of self absorption of the KMnO_4_. Therefore, 3 × 10^-4^ M KMnO_4_ was used for subsequent work (Fig. 4).

**Figure 5:**
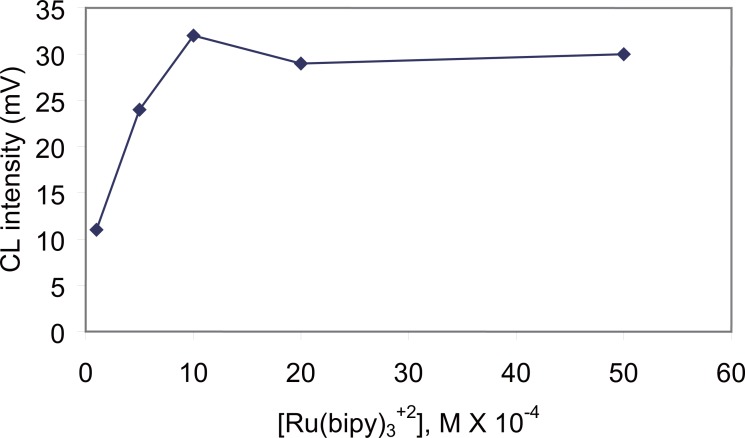
Effect of Ru(bipy)32+ concentration on the CL intensity of carvedilol (0.4 µg ml^-1^).

**Effect of Ru(bipy)_3_^2+^ concentration.** The effect of the concentration of Ru(bipy)_3_^2+^ was examined in the range of 1 × 10^-4^ – 5 × 10^-3^ M. The results showed that the CL intensity increased with an increase of Ru(bipy)_3_^2+^ concentration. A 1 × 10^-3^ M was chosen as the best concentration that gave the highest CL intensity (Fig. 5).

**Figure 6:**
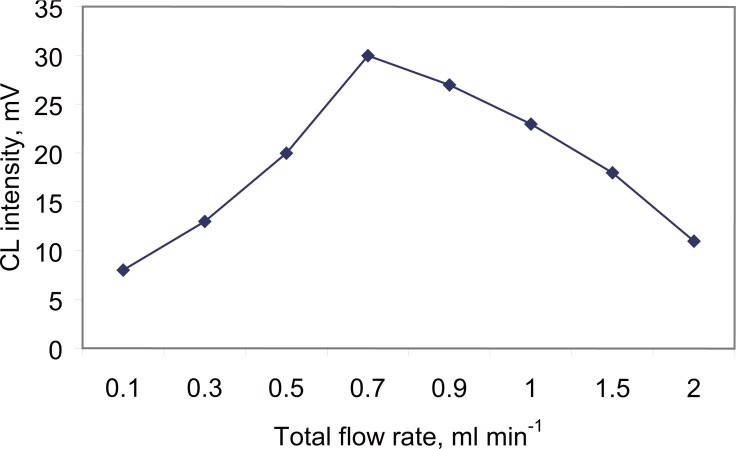
Effect of total flow rate on the CL-intensity of carvedilol (0.4 µg ml^-1^).

**Effect of Reagent flow rate.** The total flow rates of Ru(bipy)_3_^2+^ and acidified KMnO_4_ streams were varied over the range 0.1 to 2.0 ml min^-1^ with equal flows in each channel. The best total flow rate was found to be 0.7 ml min^-1^ (0.35 ml min^-1^ for each channel) as shown in Fig. 6

**Effect of sample volume.** The sample volume was varied from 50–1000 µl, the CL intensity remained virtually constant through this range, but 100 µl gave the most sharp peak.

The values of both instrumental and chemical variables affecting CL intensity of carvedilol are stated in Table 1.

**Table 1:** Optimum conditions for determination of carvedilol by the proposed FI-CL method

Parameters studied	Range	Optimum

Total flow rate (ml min^-1^)	0.1-2.0	0.7
Injection volume (μl)	50-1000	100
Ru(bipy)_3_^2+^ concentration (M)	1 × 10^-4^-5 × 10^-3^	1 × 10^-3^
KMnO_4_ concentration (M)	1 × 10^-4^-5 × 10^-3^	3 ×10^-4^
H_2_SO_4_ concentration (M)	0.1-1.5	0.8

**Effect of sensitizers.** The effect of some sensitizers, including quinine sulfate, rhodamine-B and fluoresceine on the CL reaction was investigated. All these sensitizers gave no enhancement of the CL intensity.

### Method validation

Under the optimized experimental conditions, the calibration curve was linear from 0.04–1.0 µg ml^-1^. The regression equation was

$I=67.763C+0.5185\;\left(r=0.9992,n=7\right)$

where I = intensity of the peak height (mV). C = concentration of carvedilol in µg ml^-1^.

Statistical evaluation of the regression parameters gave the following values: standard deviation of the slope (S_b_) 1.31, standard deviation of the intercept (S_a_) 0.42 and the residual standard deviation (S_xy_) 0.64. These small figures point out to the high precision of the method (20). The perfect linearity of the calibration curve is clear by the correlation coefficient. Limit of detection (LOD) and limit of quantitation (LOQ) were estimated from the standard deviation of the blank. A LOD (S/N=3) of 0.025 µg ml^-1^ and a LOQ of 0.035 µg ml^-1^ were obtained. The linear range of the proposed method was similar to the reported chemiluminometric method (17), but the proposed method is direct and requires simpler equipment using one pump. The accuracy of the method was tested with several synthetic samples of carvedilol with different concentrations over the range of 0.04–1.0 µg ml^-1^ (n=7).

The recovery is excellent and the results were in accordance with those given by the fluorimetric reference method (measuring the native fluorescence intensity at 356 nm with excitation at 254 nm) (14) as revealed by statistical analysis adopting Student’s *t*-test and *F*-test, where no significance difference was noticed between the two methods (Table 2).

**Table 2:** Application of the proposed FI CL method and a reference method (14) to the analysis of carvedilol in pure form and tablets

Preparation	Proposed method			Reference method (14)
	Conc.taken (µg ml^-1^)	Conc.Found (µg ml^-1^)	Recovery %	

Carvedilol (bulk)	0.05	0.0505	101.00	99.29
	0.1	0.0990	99.00	101.66
	0.2	0.2010	100.50	99.29
	0.4	0.3920	98.00	99.52
	0.6	0.6098	101.63	99.76
	0.8	0.7936	99.20	99.29
	Mean ± SD	-	99.87 ± 1.53	99.79 ± 1.03
	t	-	0.10 (2.23)	
	F	-	2.17 (5.05)	
Dilatrend tablets (25 mg carvedilol/tablet)	0.08	0.0797	99.59	-
	0.1	0.1000	100.00	-
	0.2	0.1993	99.66	-
	0.5	0.4976	99.52	-
	0.8	0.7981	99.76	-
	1.0	0.9982	99.82	-
	Mean ± SD	-	99.73 ± 0.158	99.70 ± 0.143^a^
	t	-	0.33 (2.26)	
	F	-	1.23 (6.26)	

a (n=5). Figures in parentheses are the tabulated t- and F-values at (*p*=0.05) (20).

Inter and intraday precision were assessed by analyzing three plasma samples containing different concentrations of the drug (Table 3) on the same day and for six days, respectively. The relative standard deviations were less than 5.9% and 7.2%, respectively.

**Table 3:** Inter and Intra-assay precision and accuracy for carvedilol in spiked human plasma

Conc. Added (µg ml^-1^)	Conc. Found (µg ml^-1^)	RSD %	Recovery^a^ %

Inter-assay			
0.1	0.096	3.4	96.36
0.4	0.408	4.5	102.00
0.8	0.835	5.9	104.43
Intra-assay			
0.1	0.098	6.3	98.45
0.4	0.402	7.2	100.50
0.8	0.822	2.2	102.70

a Results are the mean of 6 determinations.

Specificity of the proposed method for the determination of 0.4 µg ml^-1^ carvedilol was examined in the presence of excipients (sucrose, lactose, povidone, magnesium stearate) in the same ratio of those used in tablet dosage form. These excipients had no effect on carvedilol CL response.

### Application

The proposed CL method was applied to determine carvedilol in tablets. Table 2 shows that the results obtained are in good agreement with the claimed value and with those given by the reference method (14) as revealed by statistical analysis adopting Student’s *t*-test and *F*-test where no significant difference was noticed between the two methods.

A standard addition procedure was carried out in order to test the recovery of the proposed method. Thus, carvedilol concentration of 0.5 µmg ml^-1^ was added to known samples of tablets. After measuring the CL intensity, the recovery of each spiked sample was calculated (Table 4).

**Table 4:** Application of the standard addition technique to the proposed FI-CL method in Tablets

Sample No.	Sample content (µg ml^-1^)	Carvedilol (µg ml^-1^)		Recovery^a^ %
		Added	Found	

1	0.1	0.5	0.494 ± 0.032	98.82
2	0.2	0.5	0.479 ± 0.010	95.90
3	0.3	0.5	0.509 ± 0.042	101.77
4	0.4	0.5	0.509 ± 0.036	101.77
Mean (± S.D.)	-	-	-	99.57 ± 2.43

a Each value is the mean of five determinations.

The high sensitivity of the proposed method allowed the method to be applied to the *in-vitro* determination of the drug in spiked human plasma. A prior deprotenization step was necessary before application of the method. A calibration graph was first obtained by spiking plasma sample with carvedilol in the range 0.05-1.0 µg ml^-1^. Liner regression analysis of the data gave this equation:

$I=54.325C+1.0384\;\left(r=0.9985\right)$

where I is intensity of the peak height (mV) and C is the concentration of carvedilol in plasma in µg ml^-1^. The lower limit of detection was found to be 0.04 µg ml^-1^. The results of analysis of 3 spiked samples of plasma are presented in Table 3.

### Interference

Ampicillin, which is often co-adminstered with carvedilol to treat heart trouble in the clinic (13), and hydrochlorothiazide, which is co-formulated with carvedilol, did not interfere with the assay of carvedilol as they have no CL – response under the proposed conditions.

### Suggested CL mechanism

Ru(bipy)_3_^2+^ CL has proven to be a very sensitive detection system for compounds which contains amine (18). Thus the proposed reaction mechanism is thought to involve the oxidation of Ru(bipy)_3_^2+^ and the N-heterocyclic nitrogen of the carbazole moiety and or the nitrogen of secondary amino group in the side chain of carvedilol by KMnO_4_. The oxidation product of amine undergoes deprotonation to form a radical. This reduces the Ru(bipy)_3_^2+^ to the excited state that subsequently emits light.

$\begin{array}{c} \mathrm{Carvedilol}+{\mathrm{KMnO}}_{4}\to {\mathrm{Carvedilol}}^{•+}+{\mathrm{Mn}}^{2+}+{4H}_{2}O\\ {\mathrm{Carvedilol}}^{•+}\to {\mathrm{Carvedilol}}^{•}+H^{+}\\ \mathrm{Ru}{{\left(\mathrm{bipy}\right)}_{3}}^{3+}+{\mathrm{Carvedilol}}^{•}+H_{2}O\to {\left[\mathrm{Ru}{{\left(\mathrm{bipy}\right)}_{3}}^{2+}\right]}^{*}+\mathrm{Carvedilol}+H^{+}\\ {\left[\mathrm{Ru}{{\left(\mathrm{bipy}\right)}_{3}}^{2+}\right]}^{*}\to {\mathrm{Ru}{\left(\mathrm{bipy}\right)}_{3}}^{2+}+\mathrm{Light} \end{array}$

## CONCLUSION

A flow-injection CL method has been established for the determination of carvedilol based on the reaction of the drug with Ru(bipy)_3_^2+^ and KMnO_4_ in sulfuric acid medium. The method has the merits of high sensitivity, selectivity, precision, wide linear range and a short analytical time. Consequently, the method can be used for carvedilol determination in pharmaceuticals and human plasma in presence of ampicillin without prior separation, as there is no interference from ampicillin on the determination of carvedilol by the proposed CL-method. The developed method offers a similar linear range comparatively to the reported chemiluminometric method (17), but the proposed method is simpler; using a single pump and it could be used for the determination of carvedilol in presence of ampicillin and in human plasma.
